# Long-Term Effectiveness of Stress Management at Work: Effects of the Changes in Perceived Stress Reactivity on Mental Health and Sleep Problems Seven Years Later

**DOI:** 10.3390/ijerph15020255

**Published:** 2018-02-03

**Authors:** Raphael M. Herr, Amira Barrech, Natalie Riedel, Harald Gündel, Peter Angerer, Jian Li

**Affiliations:** 1 Institute of Occupational, Social and Environmental Medicine, Centre for Health and Society, Faculty of Medicine, University of Düsseldorf, Universitätsstrasse 1, 40225 Düsseldorf, Germany; amira.barrech@uni-ulm.de (A.B.); Natalie.Riedel@uni-duesseldorf.de (N.R.); Peter.Angerer@uni-duesseldorf.de (P.A.); Jian.Li@uni-duesseldorf.de (J.L.); 2 Mannheim Institute of Public Health, Social and Preventive Medicine, Medical Faculty Mannheim, Heidelberg University, Ludolf-Krehl-Strasse 7-11 68167 Mannheim, Germany; 3 Department of Psychosomatic Medicine and Psychotherapy, Faculty of Medicine, University of Ulm, Albert-Einstein-Allee 23, 89081 Ulm, Germany; Harald.Guendel@uniklinik-ulm.de; 4 Department of Social Epidemiology, Institute of Public Health and Nursing Research, University of Bremen, Grazer Str. 4, 28195 Bremen, Germany

**Keywords:** stress reactivity, work stress, mental health, sleep problems, longitudinal, stress management intervention, long-term effectiveness

## Abstract

The reduction of stress reactivity resulting from stress management interventions prevents disorders and improves mental health, however, its long-term sustainability has been little examined. The objective of this study was, therefore, to determine the effectiveness of a stress management intervention, designed to improve stress reactivity, for mental health and sleep problems seven years later, using longitudinal data from 101 male industrial workers. Linear regressions estimated the adjusted effects of the changes in stress reactivity in general as well as in its six subdimensions (work overload, social conflict, social stress, failure at work, and anticipatory and prolonged reactivity) on depression, anxiety, and sleep problems seven years later. The improvement of the prolonged reactivity had positive effects on depression, anxiety, and sleep problems (unstandardized regression coefficients [*Bs*] ≥ 0.35, all *p*-values ≤ 0.01). Depression and sleep problems were further improved by a reduction of the reactivity to social conflicts (*Bs* ≥ 0.29, *p*-values < 0.05), and an improvement in the overall reactivity score positively influenced sleep problems (*B* = 0.07, *p* = 0.017). In conclusion, the improvement of stress reactivity resulting from a work stress intervention was effective and generally long-lasting in preventing mental health and sleep problems. The reduction of the prolonged reactivity seems of particular importance and efficient in inhibiting negative stress manifestations.

## 1. Introduction

Ample evidence indicates chronic stress as a risk factor for physical and mental health [1]. It is for example known that chronic stress at work manifests in poor mental health, such as depression and anxiety [2,3], as well as in in sleep problems [4]. However, individual differences in the reactions to stress (also referred to as stress reactivity) are seen to be critical in understanding the stress effects on health, since they can influence an individual’s susceptibility to develop chronic diseases [5].

Stress reactivity has been introduced as a psychosocial construct defining to what extent a person responds to stressors with immediate, intense, and long-lasting stress reactions [6,7]. Stress reactivity is seen as a relative stable disposition of a person which, however, could change within a particular range depending on current or previous exposures to stress and which is seen to be sensitive to stress management [6,7,8,9]. The stress reactivity scale comprises a variety of stress situations, i.e., reactivity to work overload, social conflicts, social stress, failure, and anticipatory and prolonged reactivity [6,10]. Individual differences in stress reactivity have been linked to psychological and physiological functioning, including anxiety, depression, fatigue, sleep quality, physical complaints, negative health behaviour, and cortisol response to psychosocial stress [5,6,10,11]. Thus, the reduction in stress reactivity appears promising to prevent or buffer the negative consequences of stressors on health.

Workplace interventions appear successful in preventing the negative consequences of work stress. A recent meta-review provided compelling evidence for the positive effects of work-related interventions in maintaining mental health and facilitating recovery, which certifies stress management interventions through a strong previous evidence for symptom reduction [12]. However, to our best knowledge, little is known as to whether an improved stress reactivity as a result of a stress management intervention leads to favourable long-term health effects [13].

Stress management interventions might diminish the negative consequences of work stress by reducing the stress reactivity. Based on the work stress theory and by using modified techniques of group psychotherapy, our research group conducted a stress management intervention at the workplace to reduce stress reactivity [9]. The intervention comprised a tailored group-orientated stress prevention seminar based on a previous successful worksite stress prevention programme [14] that applied psychodynamic, conflict- and emotion-focused principles, but also included elements of behavioural cognitive techniques. A randomized controlled trial confirmed the effective diminishment of stress reactivity and sympathetic activation shortly after the stress management intervention. Overall, the stress reactivity, but also the reactivity in subdimensions (work overload, social conflict, social stress, failure at work, and anticipatory and prolonged reactivity), improved considerably, with the greatest effect in prolonged stress reactivity (effect size [Cohen’s *d*] = 0.465; 95%; confidence interval 0.126–0.803). This enhanced stress reactivity was associated with improved mental health within a one year period [9].

Whereas our previous findings indicate short-term effects of the changes in stress reactivity on mental health, little is known about the long-term effectiveness of stress management interventions and the responsible stress reactivity dimension. Thus, our participants from that previous study were followed up to investigate this question. This study, accordingly, aimed to examine the long-term effectiveness of the stress management intervention by estimating the effects of the changes in stress reactivity and in its subdimensions on depression, anxiety, and sleep problems seven years later.

## 2. Materials and Methods

### 2.1. Study Population

The participants of a randomized controlled trial with a waiting control group design, conducted in an international manufacturing plant in Southern Germany, were followed up seven years after all participants had received the intervention. The results and details of the randomized controlled trial can be found elsewhere [9]. In short, all eligible persons (industrial employees in production line with leadership responsibility; between 18 and 65 years old; no surgery or severe disease potentially leading to more than 30 sick leave days; *N* = 262) were invited to a baseline examination (2006). A total of 174 (66%) individuals participated in the stress management intervention either in 2006 (intervention group) or 2007 (waiting control group). The intervention consisted of a two-day basic seminar with eight teaching units and two half-day booster sessions within the following three to six months. The purpose of the intervention was to increase awareness of the typical stressors at the workplace and to improve the perception of the awareness of and the insight into stress situations at the workplace. Furthermore, tools to deal better with typical stressful situations, such as work overload, social conflicts, problems with social evaluation, and failure at work, were provided. In addition, the participants were encouraged to identify and strengthen individual resources (e.g., social networking and social support) and to exercise to recover from work [9]. This stress management intervention has shown to be effective in the direct improvement of stress reactivity [9] and to influence the long-term progression of mental health and work stress perception significantly [15]. However, so far nothing can be said about the combination of both, i.e., how the degree of change in stress reactivity affects mental health and sleep problems in the long run, which was therefore the aim of this study.

A total of 131 (75%) participants were followed up in 2008, and 102 participants in 2015 (59%). The data used for this study were gathered from questionnaires administered to the participants in 2006 (i.e., before the intervention), in 2008 (i.e., after the intervention in both groups), and in 2015. This study included all male persons with valid data on outcome, exposure, and covariates as measured in 2006, 2008, and 2015 (*N* = 101). A written informed consent was obtained, and information was provided according to the Declaration of Helsinki; the ethical committee of the University of Ulm approved the study (02/15).

### 2.2. Measures

Stress reactivity was measured by a 29-item Stress Reactivity Scale (SRS) [6], assessing general stress reactivity (Cronbach’s α: 2006 = 0.926; 2008 = 0.939; range*_low–high_* 29–87), and stress reactivity in the following specific domains: work overload (5 items; Cronbach’s α: 2006 = 0.791; 2008 = 0.864; range*_low–high_* 5–15), social conflict (6 items; Cronbach’s α: 2006 = 0.754; 2008 = 0.796; range*_low–high_* 6–18), social stress (5 items; Cronbach’s α: 2006 = 0.693; 2008 = 0.762; range*_low–high_* 5–15), failure at work (5 items; Cronbach’s α: 2006 = 0.745; 2008 = 0.759; range*_low–high_* 5–15); also, two scales evaluated stress reactivity before (anticipatory reactivity, 4 items; Cronbach’s α: 2006 = 0.730; 2008 = 0.791; range*_low–high_* 4–12) and after (prolonged reactivity, 4 items; Cronbach’s α: 2006 = 0.794; 2008 = 0.737; range*_low–high_* 4–12) stressful events.

Depressive symptoms and anxiety were assessed by the Hospital Anxiety and Depression Scale [16]. Seven items assessed depression (Cronbach’s α: 2006 = 0.794; 2015 = 0.878; range*_low–high_* 0–21) and anxiety (Cronbach’s α: 2006 = 0.743; 2015 = 0.853; range*_low–high_* 0–21).

Sleep problems were assessed similarly to other studies [17] and following parts of the Diagnostic and Statistical Manual of Mental Disorders (DSM-IV) criteria for primary insomnia complaints (i.e., difficulty initiating or maintaining sleep, or non-restorative sleep [18]) by two items, capturing complaints concerning difficulties in falling asleep and difficulties to sleep through the night (not at all; barely; to some degree; considerably; heavy). A sum score was computed ranging from two (for no sleep problems) to ten (indicating heavy sleep problems) (Cronbach’s α: 2006 = 0.644; 2015 = 0.666).

The covariates measured in this study included sociodemographic and socioeconomic factors, participation and year of stress management intervention, work characteristics, lifestyle factors, and physical and psychological conditions at baseline in 2006. Partnership and education were classified into two categories (married or having life partner versus not; medium to high versus low education [9 years or less], respectively). The work characteristics comprised shift work (yes versus no) and span of control (personnel responsibility for ≤10; 11–30; 31–50; >50 persons). Lifestyle factors included smoking behaviour (no or former smoker versus smoker), and physical activity per week (no activity; ≤1 h; 1–3 h; >3 h). The body mass index (BMI) was calculated based on height and weight, assessed by standard anthropometric methods. Chronic disease (no versus yes) was defined as a past or current serious illness or the suffering from a mental, cardiovascular, or lung disease. The presence of stressful life events (no versus yes) was assessed by applying ten items of the list of threatening events [19], from which two unfitting items regarding unemployment were excluded.

### 2.3. Statistical Analysis

Changes in the perceived stress reactivity were defined as the difference between the baseline stress reactivity scores (2006) and the stress reactivity scores after the stress management intervention (2008) by subtracting the baseline values from the follow-up values. In consequence, a positive value indicates an increase in stress reactivity, while a negative value represents a decrease. Linear regression models were used to estimate the effects of the stress reactivity change scores on depression, anxiety, and sleep problems in 2015. Unstandardized (*B*) and standardized (*β*) regression coefficients, as well as the proportion of explained variance (*R*^2^) were reported. To omit floor or ceiling effects, all analyses were adjusted for the baseline values of the specific stress reactivity score and the outcome variable (i.e., depression, anxiety, or sleep problems). For each outcome, four hierarchical regression models were calculated: the first models were adjusted for age, education, partnership, and participation in and year of the stress management intervention; the second models were additionally adjusted for work characteristics (shift work, personnel responsibility); the third models also included lifestyle factors (smoking, physical activity, BMI), and the last models additionally took chronic diseases and critical life events into account. The analyses were performed using SPSS (Version 21, SPSS Inc., Chicago, IL, USA).

## 3. Results

### 3.1. Participant Characteristics

The participant characteristics at baseline are presented in Table 1. The mean age was 41.6 years (SD = 7.43). Most of the participants had a lower educational level (9 years or less; 57%), and were married or lived with a partner (92%). The stress management intervention seminars were almost evenly distributed in the years 2006 and 2007. The majority of participants did shift work (58%), and—with regard to healthy lifestyles—were nonsmokers (74%) and engaged in at least one hour of physical activity per week (79%). The average BMI was 28 kg/m^2^ (SD = 4.01). Nearly 60% of the participants reported some chronic disease and 84% reported at least one important life event in the last year.

### 3.2. Overview of the Main Variables

Table 2 presents the descriptive statistics (first two rows) and the bivariate correlations between the main variables. The correlations between the stress reactivity dimensions were modest to high. Regarding mental health and sleep problems, especially the change in prolonged reactivity showed a notable significant association.

### 3.3. Effects of the Changes in Stress Reactivity on Mental Health

Linear regressions revealed that a reduction in stress reactivity to social conflicts and in prolonged stress reactivity had a positive effect on depression scoring (i.e., lower depression scoring) seven years later (Model 4: *B_social conflicts_* = 0.41; *p* = 0.048; *R*^2^ = 19%; *B_prolonged reactivity_* = 0.80; *p* = 0.003; *R*^2^ = 24%; Table 3). Two further effects were significant in trend: the decline in total score and in failure at work dimensions (Model 4: *B_overall reactivity_* = 0.09; *p* = 0.103; *R*^2^ = 18%; *B_failure_*_at work_ = 0.49; *p* = 0.084; *R*^2^ = 18%; Table 3). Regarding anxiety, the improvement in the prolonged reactivity had likewise the strongest effect (Model 4: *B_prolonged reactivity_* = 0.62; *p* = 0.012; *R*^2^ = 25%; Table 4). The effects of stress reactivity to social conflicts and failure at work were significant in trend (Model 4: *B_social conflicts_* = 0.33; *p* = 0.080; *R*^2^ = 23%; *B_failure_*_at work_ = 0.46; *p* = 0.070; *R*^2^ = 21%; Table 4).

### 3.4. Effects of the Changes in Stress Reactivity on Sleep Problems

The improvement in the overall stress reactivity score, as well as in the work overload, the social conflicts, and the prolonged reactivity subdimensions positively affected sleep quality seven years later (Model 4: *B_overall score_* = 0.07; *p* = 0.017; *R*^2^ = 17%; *B_social conflicts_* = 0.29; *p* = 0.005; *R*^2^ = 18%; *B_prolonged reactivity_* = 0.35; *p* = 0.010; *R*^2^ = 17%; Table 5). In the full adjusted model, the effect of the work overload was marginally not significant (*B*_work overload_ = 0.23; *p* = 0.059; *R*^2^ = 19%; Table 5).

## 4. Discussion

The current study documents the long-term effects of the changes in stress reactivity on mental health and sleep problems. Depression, anxiety, and sleep problems were significantly diminished seven years later especially when the prolonged stress reactivity was improved during the intervention. In addition, a reduced reactivity to social conflicts had a positive effect on depression and sleep problems, while the latter was also positively affected by a change in the overall stress reactivity score.

The strongest effect on mental health and sleep problems was found for changes in the prolonged stress reactivity. This dimension captures the difficulty to recover from a high work load (i.e., post-stress) [5]. An impaired recovery might constitute a link between stressful work characteristics and long-term effects on health, like sleep problems and psychosomatic complaints [20,21,22]. A repeated insufficient recovery from work is assumed to cumulate into fatigue leading to health deterioration, possibly caused by a prolonged increased physiological activity leading to allostatic load (i.e., bodily wear and tear) [20,22,23,24,25,26,27]. An improvement in the prolonged reactivity might thus promote recovery from work and prevent negative health consequences.

The observation that changes in stress reactivity had strong effects on sleep problems is in line with empirical evidence showing stress to be related to impaired sleep [28,29]. As sleep problems are considered to be a marker of prolonged stress, it is not surprising that in particular an increase in the prolonged reactivity influences sleep quality negatively [28]. Furthermore, the effect of the reactivity to work overload is in line with empirical findings, revealing high work demands to have a robust impact on sleep disturbances [4]. As impaired sleep constitutes a gateway to manifold diseases, including myocardial infarction, autoimmune diseases, and depression [30,31,32], it is highly valuable to prevent sleep problems by reducing stress reactivity.

A reduced reactivity to social conflicts had positive effects on depression and sleep problems; the effects on anxiety did not reach the threshold of significance (*p* = 0.080). The reactivity to social conflicts encompasses situations in which the participant feels affected, annoyed, or upset in response to social conflict, or disliked, criticised, and rejected. Such a social negativity increases the psychological distress, which, however, might be buffered by emotional regulation [33], possibly learned in stress management interventions [9].

The changes in stress reactivity predicted mental health and sleep problems independently from potential influencing factors. The analyses were controlled for sociodemographic and -economic factors, as well as for work characteristics, chronic disease, and life events. Further, the associations did not seem to be mediated by lifestyle factors, as the adjustments for smoking, physical activity, and BMI did not attenuate the effect sizes. This suggests that these factors are unlikely to provide a major explanation for our findings.

Stress reactivity refers to the disposition of a person to respond to stressors with immediate, strong, and long-lasting stress reaction and is assumed to be a vulnerable factor for the development of diseases [6,7]. It is thought to be both stable and variable [10], and can be changed as a result of stress management interventions [9]. Such a change, e.g., due to learned coping mechanisms like the detection of strain symptoms and the techniques to deal with them, might buffer the negative health consequences of a stressful work experience.

This study possibly informs about the effectiveness of the different components of stress management interventions. The work stress management training applied here—which is based on the work stress theory—used modified techniques of group psychotherapy and aimed to improve the following factors: identification and strengthening of social networking and social support, early identification of typical work stress situations, and techniques to deal with such stressful situations, including psychological detachment from work [9]. Particularly the latter appeared to be relevant as we found that the strongest effects on mental health and sleep problems corresponded to changes in prolonged stress reactivity, capturing the difficulty to recover following a high workload. Against this background and in combination with further empirical evidence showing that stress management interventions also have the potential to reduce burnout and enhance work well-being, as well as stress recovering [34,35], it appears appropriate to conclude that a recovery intervention in terms of psychological detachment from work is a successful component of the stress management intervention. With respect to practical implications, training focusing on practices to deal with stress and on detachment from work stress seems especially advisable. Such training might comprise specific tools to efficiently recover from stress factors at work. In this context, the model of “psychosomatic consultation in the workplace” (PCIW) appears also relevant. PCIW is an innovative concept to provide early psychotherapeutic consultation in the workplace through a close cooperation between company-based occupational health physicians and external psychotherapeutic consultants. PCIW therefore can provide early, easy, and fast first access to mental health care for employees [36,37,38].

Some limitations must be taken into consideration. First, the sample was restricted to men, and a generalization to women is still to be determined. Second, albeit 61% represents a good follow-up rate, a selection bias cannot be ruled out, e.g., a healthy worker effect may have occurred, whereby those with highest stress reactivity and potentially related ill health are more likely to have left employment. This might have restricted the measurement range and potentially led to an underestimation of the true associations. However, a comparison of the study sample with the persons which could not be followed up (drop-out analysis), revealed no significant differences in demographic, lifestyle factors, stress reactivity, or change in stress reactivity (*p*-values > 0.10), pointing against a strong bias. Despite this and the comprehensive set of potential covariates considered, the possibility of alternative explanations cannot be fully eliminated. Third, the stress reactivity was assessed by self-report. Such an assessment of perceived reactivity is, however, assumed to be a valuable and convenient indicator of stress reactivity [39]. A comparison of the here-applied German with the English version of the stress reactivity scale is however limited because of a different factor structure (six versus five factors) and number of items (29 versus 23 items) [40]. Fourth, multiple testing might have inflated the overall Type I error rate. In consequence, a more conservative level of significance might be applied, and, especially, the associations with *p*-values < 0.01 (i.e., two asterisks in Table 3, Table 4 and Table 5) should be considered to be relevant.

## 5. Conclusions

In conclusion, an innovative aspect of the present study is the examination of the long-term effectiveness of the perceived stress reactivity improvement by stress interventions at work: it positively affected mental health and sleep problems seven years later. Stress management interventions aimed at stress reactivity reduction—especially regarding the prolonged reactivity—represent a promising target to prevent negative stress sequelae.

## Figures and Tables

**Table 1:** Sample Characteristics at Baseline (*N* = 101).

		% or Mean	*N* or SD
Age (years)		41.63	7.43
Education	Medium to high	42.6%	43
	Low (≤9 years)	57.4%	58
Married or having life partner		92.1%	93
Participation in SMI		90.1%	91
Year of SMI	2006	42.9%	39
	2007	57.1%	52
Shift work		58.4%	59
Personnel responsibility	≤10 persons	23.8%	24
	11–30 persons	23.8%	24
	31–50 persons	26.7%	27
	>50 persons	25.7%	26
Smoking behaviour	No or former smoker	74.3%	75
	Smoker	25.7%	26
Physical activity/week	No	20.8%	21
	≤1 h	11.9%	12
	>1–3 h	43.6%	44
	>3 h	23.8%	24
BMI		28.23	4.01
Any chronic disease		59.4%	60
Any important life event		84.2%	85

SMI = stress management intervention; BMI = body mass index.

**Table 2:** Descriptive Statistics and Correlations between the Main Variables (*N* = 101).

	∆ Overall Reactivity	Overall Reactivity	∆ Work Overload	Work Overload	∆ Social Conflicts	Social Conflicts	∆ Social Stress	Social Stress	∆ Failure at Work
Mean	−4.39	54.86	6.29	1.73	9.46	2.06	6.54	1.81	7.39
Std. Deviation	7.97	10.76	2.13	0.47	2.43	0.42	2.22	0.47	1.85
Overall reactivity	−0.310 **								
∆ Work Overload	0.462 **	0.515 **							
Work overload	−0.164	0.824 **	0.571 **						
∆ Social Conflicts	0.359 **	0.634 **	0.622 **	0.570 **					
Social Conflicts	−0.328 **	0.842 **	0.358 **	0.581 **	0.580 **				
∆ Social stress	0.494 **	0.466 **	0.576 **	0.413 **	0.682 **	0.328 **			
Social stress	−0.157	0.803 **	0.447 **	0.635 **	0.564 **	0.618 **	0.588 **		
∆ Failure at work	0.339 **	0.620 **	0.664 **	0.558 **	0.711 **	0.503 **	0.556 **	0.448 **	
Failure at work	−0.193	0.804 **	0.423 **	0.568 **	0.541 **	0.647 **	0.419 **	0.602 **	0.583 **
∆ Anticipatory reactivity	0.451 **	0.495 **	0.651 **	0.500 **	0.563 **	0.287 **	0.596 **	0.478 **	0.589 **
Anticipatory reactivity	−0.281 **	0.835 **	0.440 **	0.719 **	0.501 **	0.622 **	0.353 **	0.636 **	0.512 **
∆ Prolonged reactivity	0.568 **	0.191	0.442 **	0.144	0.405 **	0.146	0.425 **	0.168	0.409 **
Prolonged reactivity	−0.366 **	0.670 **	0.216 *	0.424 **	0.260 **	0.519 **	0.115	0.328 **	0.360 **
Depression	0.114	0.176	0.104	0.227 *	0.233 *	0.116	0.178	0.078	0.254 *
Anxiety	0.065	0.285 **	0.228 *	0.289 **	0.299 **	0.260 **	0.192	0.103	0.291 **
Sleep Problems	0.156	0.183	0.313 **	0.304 **	0.243 *	0.090	0.183	0.094	0.202 *
	Failure at work	∆ Anticipatory reactivity	Anticipatory reactivity	∆ Prolonged reactivity	Prolonged reactivity	Depression	Anxiety	Sleep Problems	
Mean	2.02	5.16	1.97	4.33	1.72	4.66	5.90	4.11	
Std. Deviation	0.42	1.86	0.51	1.63	0.55	4.03	3.79	1.85	
Overall reactivity									
∆ Work Overload									
Work overload									
∆ Social Conflicts									
Social Conflicts									
∆ Social stress									
Social stress									
∆ Failure at work									
Failure at work									
∆ Anticipatory reactivity	0.421 **								
Anticipatory reactivity	0.579 **	0.377 **							
∆ Prolonged reactivity	0.192	0.482 **	0.162						
Prolonged reactivity	0.483 **	0.304 **	0.466 **	0.099					
Depression	0.136	0.135	0.125	0.305 **	0.159				
Anxiety	0.199 *	0.181	0.233 *	0.260 **	0.277 **	0.748 **			
Sleep Problems	0.111	0.180	0.084	0.261 **	0.183	0.373 **	0.501 **		

∆ = change, *N* = 101. *p* ≤ 0.1; * *p* ≤ 0.05; ** *p* ≤ 0.01.

**Table 3:** Effects of the Changes in Stress Reactivity on Depression (*N* = 101).

	Depression											
	Model 1			Model 2			Model 3			Model 4		
	*B*	*β*	*R^2^*	*B*	*β*	*R^2^*	*B*	*β*	*R^2^*	*B*	*β*	*R^2^*
Overall reactivity	0.091	0.180	0.142	0.091	0.180	0.144	0.097	0.191	0.175	0.092	0.182	0.180
Work overload	−0.186	−0.086	0.132	−0.180	−0.084	0.133	−0.118	−0.055	0.158	−0.140	−0.065	0.168
Social conflicts	0.393 *	0.203	0.149	0.394 *	0.204	0.151	0.415 *	0.215	0.182	0.412 *	0.214	0.189
Social stress	0.328	0.153	0.133	0.343	0.160	0.136	0.362	0.169	0.166	0.343	0.160	0.171
Failure at work	0.489	0.196	0.146	0.483	0.193	0.146	0.461	0.185	0.172	0.491	0.197	0.183
Anticipatory reactivity	0.147	0.069	0.117	0.149	0.071	0.119	0.138	0.065	0.147	0.088	0.042	0.155
Prolonged reactivity	0.776 **	0.423	0.204	0.797 **	0.434	0.209	0.812 **	0.443	0.236	0.795 **	0.433	0.238

Model 1: adjusted for outcome and exposure at baseline, age, education, partnership, participation in and year of stress management intervention. Model 2: model 1 + adjusted for work characteristics (shift work, personnel responsibility). Model 3: model 2 + adjusted for lifestyle (smoking, physical activity, BMI). Model 4: model 3 + adjusted for chronic diseases and life events. *p* ≤ 0.1; * *p* ≤ 0.05; ** *p* ≤ 0.01.

**Table 4:** Effects of the Changes in Stress Reactivity on Anxiety (*N* = 101).

	Anxiety											
	Model 1			Model 2			Model 3			Model 4		
	*B*	*β*	*R^2^*	*B*	*β*	*R^2^*	*B*	*β*	*R^2^*	*B*	*β*	*R^2^*
Overall reactivity	0.073	0.153	0.177	0.073	0.153	0.178	0.080	0.168	0.220	0.084	0.176	0.228
Work overload	0.079	0.039	0.164	0.076	0.038	0.164	0.176	0.087	0.209	0.172	0.085	0.216
Social conflicts	0.321	0.206	0.177	0.325	0.179	0.185	0.333	0.183	0.222	0.334	0.184	0.228
Social stress	0.323	0.160	0.140	0.321	0.159	0.140	0.351	0.174	0.183	0.373	0.185	0.190
Failure at work	0.489 *	0.208	0.170	0.497 *	0.212	0.171	0.468	0.199	0.208	0.463	0.197	0.211
Anticipatory reactivity	0.131	0.066	0.146	0.137	0.069	0.148	0.121	0.061	0.196	0.142	0.071	0.202
Prolonged reactivity	0.558 *	0.324	0.203	0.563 *	0.327	0.203	0.592 **	0.343	0.238	0.623 **	0.361	0.246

Model 1: adjusted for outcome and exposure at baseline, age, education, partnership, participation in and year of stress management intervention. Model 2: model 1 + adjusted for work characteristics (shift work, personnel responsibility). Model 3: model 2 + adjusted for lifestyle (smoking, physical activity, BMI). Model 4: model 3 + adjusted for chronic diseases, and life events. *p* ≤ 0.1; * *p* ≤ 0.05; ** *p* ≤ 0.01.

**Table 5:** Effects of Changes in Stress Reactivity on Sleep Problems (*N* = 89).

	Sleep Problems											
	Model 1			Model 2			Model 3			Model 4		
	*B*	*β*	*R^2^*	*B*	*β*	*R^2^*	*B*	*β*	*R^2^*	*B*	*β*	*R^2^*
Overall reactivity	0.171 **	0.303	0.132	0.067 **	0.295	0.145	0.069 **	0.304	0.166	0.066 *	0.294	0.171
Work overload	0.234 *	0.237	0.141	0.217	0.219	0.151	0.251 *	0.253	0.177	0.232	0.234	0.188
Social conflicts	0.285 **	0.330	0.139	0.293 **	0.339	0.160	0.290 **	0.336	0.176	0.285 **	0.330	0.181
Social stress	0.162	0.173	0.075	0.169	0.195	0.093	0.170	0.181	0.114	0.157	0.167	0.120
Failure at work	0.153	0.133	0.067	0.151	0.131	0.083	0.146	0.127	0.102	0.164	0.143	0.114
Anticipatory reactivity	0.187	0.192	0.067	0.201	0.206	0.089	0.186	0.191	0.105	0.180	0.184	0.112
Prolonged reactivity	0.357 **	0.431	0.136	0.340 **	0.411	0.143	0.356 **	0.430	0.166	0.351 **	0.424	0.170

Model 1: adjusted for outcome and exposure at baseline, age, education, partnership, participation in and year of stress management intervention. Model 2: model 1 + adjusted for work characteristics (shift work, personnel responsibility). Model 3: model 2 + adjusted for lifestyle (smoking, physical activity, BMI). Model 4: model 3 + adjusted for chronic diseases and life events. *p* ≤ 0.1; * *p* ≤ 0.05; ** *p* ≤ 0.01.
